# Perfluorooctanesulfonate (PFOS) Induces Apoptosis Signaling and Proteolysis in Human Lymphocytes through ROS Mediated Mitochondrial Dysfunction and Lysosomal Membrane Labialization

**Published:** 2018

**Authors:** Mohammad Hadi Zarei, Seyed Farshad Hosseini Shirazi, Marjan Aghvami, Jalal Pourahmad

**Affiliations:** a *Department of Toxicology, Faculty of Pharmacy, Shahid Beheshti University of Medical Sciences, Tehran, Iran. *; b *Pharmaceutical Sciences Research Center, School of Pharmacy, Shahid Beheshti University of Medical Sciences, Tehran, Iran.*

**Keywords:** Perfluorooctanesulfonate (PFOS), Reactive oxygen species (ROS), Human lymphocyte, Cellular proteolysis, Lysosomal membrane integrity, Cytotoxicity

## Abstract

Perfluorinated compounds (PFCs) such as perfluorooctanesulfonate (PFOS) are stable chemicals that accumulate in biological matrix. Toxicity of these compounds including immunotoxicity has been demonstrated in experimental models and wildlife. Although limited number of studies examined the effects of PFOS on human lymphocytes but so far no research has investigated the complete mechanisms of PFOS cytotoxicity toward human lymphocytes. The main goal of this investigation was to find out the mechanisms underlying the cytotoxic effect of PFOS toward human lymphocytes using accelerated cytotoxicity mechanisms screening (ACMS) technique. Human lymphocytes were isolated from blood of healthy donors using Ficoll-paquePLUS standard method. Cell viability was determined following 12 h of incubation of human lymphocytes with 100-500 µM PFOS. Our results showed that IC_50_ concentration (163.5 µM) of PFOS reduced viability of human lymphocytes approximately 50% via increased ROS formation, lipid peroxidation, glutathione depletion and damage to cell sub organelles such as mitochondria and lysosomes. Besides, in this study we demonstrated involvement of cellular proteolysis and activation of caspase-3 in PFOS induced lymphocyte cytotoxicity. We finally concluded that at environmentally related concentration, PFOS can induce toxic effect toward human lymphocytes through induction of oxidative stress and damage to cell sub organelles.

## Introduction

One of the most widely used PFCs is PFOS (perfluorooctanesulfonate), synthetic chemicalapplied to produce a variety of industry and consumer products, such as refrigerants, lubricants, surfactants, and as components of pharmaceuticals, cosmetics, insecticides or ﬁre retardants for more than 50 years (1). PFOS was detected in blood samples of both wildlife and humans (2, 3). PFOS is not biodegradable and so accumulate in biological matrixes (4, 5). In serum of common people from different areas of world, PFOS concentration was about nanogram per milliliter (6) and in worker that occupationally were exposed was about microgram per milliliter (7, 8). The half-life of PFOS in humans is about 5.4 years (9).

In mice exposed to 20 mg/kg body weight per day of PFOS and PFOA for 7-10 day, the weight of thymus and spleen was decreased (10-12). These effects also have observed after long exposure of mice with these compounds (13). PFOS affect lymphocytes subpopulations and especially decrease CD4+ CD8+ double positive thymocytes (11, 12). In mice which exposed to levels signiﬁcantly below those found in occupationally exposed workers, function of lymphocyte (such as release of IgM) decreased (14). This effect was observed by Corsini *et al*. in mice exposed to levels of PFOS that are found in the general human population. PFCs also influence on differentiation and cytokine production of macrophage, related to innate immune system (15). Inﬂuenza A virus causes increased mortality in mice exposed to PFOS (16). Similar results have been observed in other species such as birds, turtles, and lizards (17). Taken together, these data show that PFCs are toxic to the immune system.

There are only limited studies in people that environmentally or occupationally were exposed to PFOS. It has been shown in a publication that especially in male children, prenatal exposure to PFOA and PFOS was positively correlated with increased IgE levels (18). Findings of this study showed the higher chance of allergic disease in children exposed to PFCs. There is a need for furtherresearches about human exposure with PFOS for gaining more reliable results. Chronic inflammatory diseases such as cancer, diabetes, and heart disease can be considered as an adverse disorder due to exposure to PFCs (17). Some studies suggest that exposure of human to PFCs cause immunosuppressive effects. Levels of immunoglobulins A and E in females, antinuclear antibodies, natural killer cell activity, and release of pro-inflammatory cytokine (TNF-α) are parts of immune system that disturbed after exposure to PFCs. High serum levels of PFCs in children may cause failure of vaccination because of immunosuppressive effect of these compounds (17).

Most research projects concern immunotoxicity of PFCs were performed in rodents (14, 15). Information about the effects of PFOS on the immune system of human are so rare, in addition a few studies assayed the adverse effect of PFOS on human lymphocytes (19). Although there are evidences showing that PFOS causes cell death in human lymphocytes, the mechanisms lead to decrease in viability of these cells have not yet been studied. So in this study we wanted to investigate the mechanisms underlying cytotoxicity of PFOS on human blood lymphocytes assess the events in cells that lead to cell death following PFOS exposure.

## Experimental

*Chemicals*

Trypan blue, 2′,7′-dichlorofuorescin diacetate (DCFH-DA), Rhodamine123, bovine serum albumin (BSA), N-(2-hydroxyethyl) piperazine-N′-(2-ethanesulfonic acid) (HEPES), acridine orange, OPA (*o*-Phthalaldehyde), NEM (N-ethylmaleimide), perfluorooctanesulfonate (PFOS) and trichloroacetic acid were purchased from Sigma-Aldrich Co. (Taufkirchen, Germany). RPMI1640 and FBS (Fetal Bovine serum) were purchased from Gibco, Life Technologies, Grand Island, NY. Ficoll-paque PLUS was obtained from Ge Healthcare Bio-Science Company.

*Ethics statement*

This investigation was performed in Shahid Beheshti University of Medical Science (SBMU) Faculty of Pharmacy and approved by Research Ethic Committee of Shahid Beheshti University of Medical Science (SBMU) (Number: IR. SBMU. RAM. REC. 1395. 7 ; Date: April 10, 2016). All participants were completely aware of this study and filled an approval form.

*Human lymphocytes isolation and treatment*

Lymphocytes were derived from healthy individual in the age range of 18 to 30 years old. Blood was obtained from 20 healthy, non-smoking donors, who did not exhibit any symptoms of infectious disease at the time of blood samples collection. Lymphocytes were isolated using ficoll paque plus standard method with some modification. Diluted blood was layered on 3 mL ficollpaque, centrifuged for 20 min at 2500 rpm and lymphocytes layer were collected, suspended in erythrocyte lysis buffer (150 mM NH4Cl, 10 mM NaHCO3, 1 mM EDTA, 183 pH 7.4), and incubated for 5 min at 37 °C. Then, PBS was added instantly, and the cells were centrifuged at 1500×g for 10 min at 20 °C. The supernatant was removed, and the cells were washed twice with RPMI with l-glutamine and 10% fetal bovine serum (FBS) at 2000×g for 7 min. The cells were resuspended in RPMI medium with l-glutamine and 10% FBS and counted using trypan blue exclusion dye. The viability of the cells was over 95% and final lymphocyte density used in the experiments was 10 × 10^6^ cells/mL. For assessment of cell viability, lymphocytes were treated with 0.0-500 µM PFOS for 12 h and for performing other tests the cells were incubated with 75, 150, and 300 µM of PFOS for 2, 4, and 6 h.

*Cell viability assay*

For measurement of cell viability, we used trypan blue exclusion dye that can penetrate membranes of dead cell and color them blue. The cells were cultured in 96-well plate in concentration of 1 × 10^4 ^cells per well. After treatment of cells with different concentration of PFOS (0, 100, 200, 300, 400 and 500 µM), same volume of cells suspension and trypan blue 0.4% were mixed. The numbers of dead and live cells were counted by use of hemocytometer and light microscope. Then percent cells viability and IC_50_ were measured.

*Measurement of ROS*

We used dichlorofluorescein diacetate (DCFH-DA) for assessment of reactive oxygen species (ROS). DCFH-DA breaks to nonfluorscent DCFH by cellular esterase. Reaction of ROS with DCFH produces fluorescent DCF. After treatment of cell with different concentration of PFOS for 2, 4 and 6 h, the cell suspension was centrifuged and the supernatant was removed and then the cells were washed with PBS. Then, the cells were treated with 500 µL of 10 µM DCFH-DA solution and incubated for 20 min in 37 °C. After washing of cells with PBS, fluorescence of DCF was measured with fluorescence spectrophotometer (Shimadzu RF5000U) at 495 and 530 nm excitation and emission wavelength (20).

*Assessment of mitochondrial membrane potential (MMP)*

Rhodamine123 was used for measurement of mitochondrial membrane potential after incubation of human lymphocytes with PFOS for different time intervals. Two, four, and six hour after treatment of lymphocytes with different concentration of PFOS, the cell suspension were centrifuged and the supernatant was removed. The human lymphocytes were incubated with 500 µL of 1 µM rhodamine123 for 15 min. After washing the cells, the fluorescence of rhodamine123 was determined at 470 nm excitation and 540 nm emission wavelength by fluorescence spectrophotometer (21). 

*Lipid peroxidation measurement*

We assessed lipid peroxidation based on the reaction of thiobarbituric acid (TBA) and malondialdehyde. Human lymphocytes were treated with different concentration of PFOS and lysed with PBS contain 2% triton. Two-hundred µL TBA reagent (TBA 0.37%, trichloroacetic acid (TCA) 15% and HCl 2.5 N) was added to 100 µL cells and heated in hot water(90 °C) for 60 min. Absorbance measured with Beckman DU-7 spectrophotometer 532 nm and concentration of TBA-MDA was determined using TBA-MDA calibration curve (22).

*Determination of proteolysis*

Reaction of OPA (*o*-Phthalaldehyde) with amino groups in presence of 2-mercaptoethanol was the basis for determination of proteolysis. The OPA solution was prepared fresh daily by combining of 50 mL of 0.1 M sodium borate-2% (w/v) NaDodS04, 80 mg of OPA (dissolved in 2.0 mL of methanol), and 0.2 mL of 2-mercaptoethanol (orethanethiol) and diluting to final volume of 100 mL with water. The Same volume of sample and 20% trichloroacetic acid was mixed and kept at 4°C for 12 h. Each sample was centrifuged and 50 µL of supernatant was blended with 1.0 mL of OPA reagent and incubated for 2 min in room temperature. After that the absorbance was determined at 340 nm in a Beckman DU-7 spectrophotometer.

*Assessment of lysosomal membrane destabilization*

Diffusion of acridine orange, a lipophilic dye that accumulates in acidic organelle of cell like lysosome, was used for assessment of lysosomal membrane integrity. Lysosomal membrane destabilization was measured 2, 4 and 6 h after incubation of human lymphocytes with different concentration of PFOS. One-hundred µL of cell suspension plus100 µL of 5 µM acridine orange incubated for 10 min in 37 °C. Finally fluorescent of diffused acridine orange was determined at 470 nm and 540 nm excitation and emission wavelength by fluorescence spectrophotometer (23).

*GSH and GSSG assessment*

Human lymphocytes were incubated with different concentration of PFOS for 2, 4, and 6 h and lysed with 0.5 mL of TCA 10%. After centrifugation at 11,000 rpm for 2 min, for GSH assay supernatant was diluted with phosphate-EDTA buffer and incubated with 100 µL of the OPT solution for 15 min at room temperature. For determination of GSSG, cells supernatant was diluted with NaOH solution (0.1N). Before incubation with OPT, Diluted supernatant was incubated with 200 µL of NEM solution for 30 min. Finally fluorescence intensity was recorded at 350 and 420 nm excitation and emission wavelength and concentration of GSH and GSSG calculated by use of GSH and GSSG calibration curve (24).

*Measurment of caspase-3 activity*

After treatment of human lymphocytes with IC_50_ of PFOS for 6 and 12 h, activity of caspase3 was determined by use of “Sigma’s caspase-3 assay kit (CASP-3-C)”. Briefly, release of *p*-nitroaniline moiety from caspase-3 substrate (Ac-DEVD-*p*NA) after hydrolysis by caspase3 is the basis of caspase3 assessment kit. Absorbance of *p*-nitroaniline at 405 nm was assessed in samples and concentration of *p*-nitroaniline was determined by use of* p*-nitroaniline calibration curve. 

*Statistical analysis*

GraphPad Prism 5 (Graphpad Software, La Jolla, CA) was used for analysis of our data. Statistical analysis of data was performed using one and two way analysis of variance followed by post-hoc Tukey and Bonferroni test. At least three independent experiments were used. *P*-value of less than 0.05 was considered as statistically significant. The results are showed as the mean ± standard error of the mean. 

## Results

*Effect of PFOS on lymphocyte viability*

**Figure 1:**
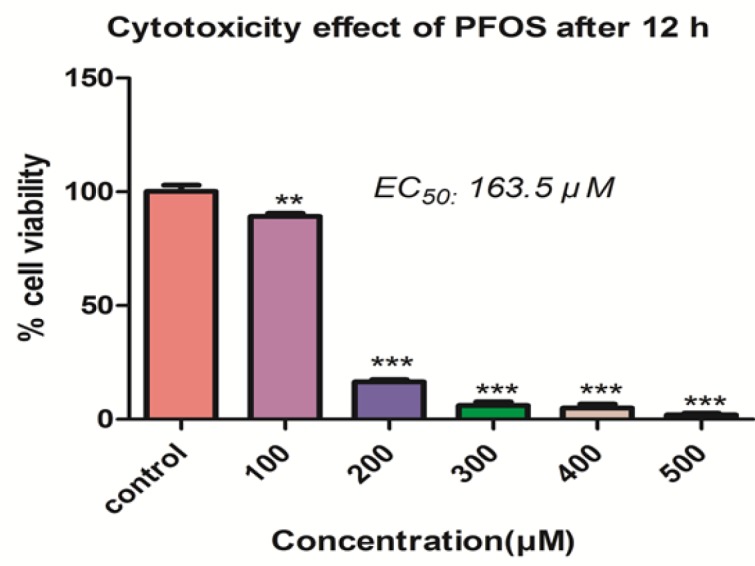
The Effect of PFOS on viability of human lymphocytes.Effect of PFOS on viability of human lymphocytes after 12 h treatment. Cell viability determined by trypan blue exclusion dye following treatment of lymphocytes with a wide range of PFOS. PFOS reduced lymphocyte viability in a dose-dependent manner and this decrease is significant at 100µM and higher concentration in comparison with control.^*^P < 0.05, ^**^P < 0.01 and ^***^P < 0.001.

The effect of PFOS on viability of human lymphocytes was demonstrated in the Figure 1. PFOS caused dose-dependent cytotoxicity on human lymphocytes and significantly (*P *< 0.05) decreased the number of viable cells at concentrations higher than 100 µM. The IC_50_ determined for PFOS toward human lymphocytes after 12 h treatment, was 163.5 µM.

*Measurement of ROS*

**Figure 2:**
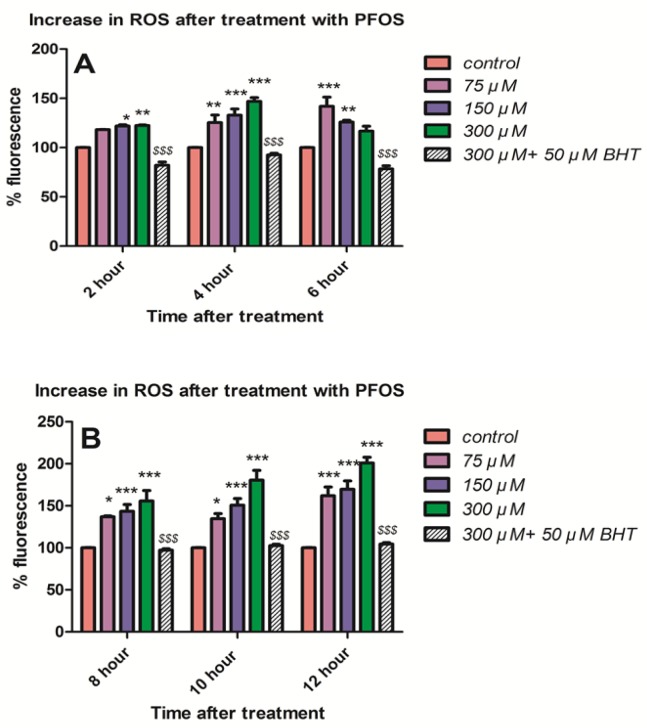
Generation ROS in isolated human lymphocyte after treatment with PFOS. ROS generation in human lymphocyte after treatment with PFOS for different time intervals. ROS generation was measured in cells using dichlorofluoresceindiacetate (DCFH-DA) and fluorescence spectrophotometer. Induction of ROS by PFOS was significant (*P <* 0.05) at concentration 150 and 300 µM at 2 h and at all concentration at 4 h and at 6 h only at concentration 75 µM in comparison with control, but ROS formation after 6 h significantly(*P <* 0.05) increased until 12 h in comparison with control. Buyhylatedhydroxytoluene (BHT), an antioxidant, inhibited ROS induction by PFOS in human lymphocytes. ^*^P < 0.05, ^**^P < 0.01 and ^***^P < 0.001.

As shown in Figure 2A, ROS formation (*P *< 0.05) was determined at 2, 4 and 6 h following treatment of human lymphocytes with different concentrations of PFOS (75, 150 and 300 µM). PFOS-induced ROS formation was measured using DCFH-DA. ROS formationat earliest time interval of 2 h was significantly (*P <* 0.05) increased only by higher concentrationsof 150 and 300 µM, nevertheless all concentrations significantly (*P *< 0.05) increased ROS formation at 4 h of treatment in human blood lymphocytes. We also followed the changes in lymphocyte ROS generation during the incubation with PFOS. Following a considerable fall at 6h, ROS formation was significantly (*P <* 0.05) increased again at 8 h, and trend of increase at ROS generation remained until 10 and 12 h of incubation with PFOS (Figure 2B). Preincubation of isolated human lymphocytes with an antioxidant, buyhylatedhydroxytoluene (BHT), prevented ROS formationinduced by different concentrations of PFOS.

*Mitochondrial membrane potential*

**Figure 3:**
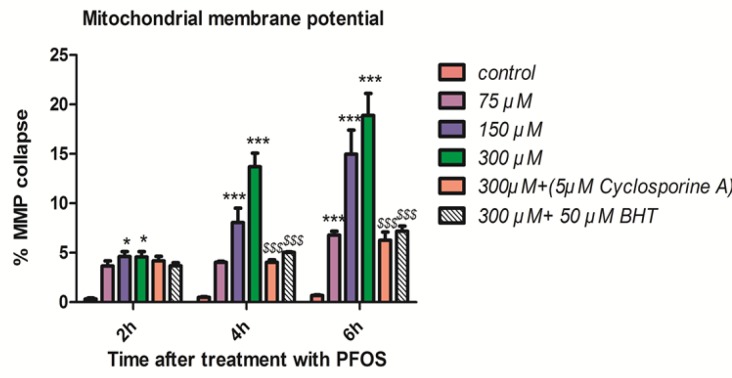
Collapse of mitochondrial membrane potential (MMP) in human lymphocytes following PFOS treatment.PFOS-induced collapse of mitochondrial membrane potential (MMP) in human lymphocytes. MMP was assessed at 2, 4 and 6 h following incubation of lymphocytes with PFOS. Two hours after treatment of human lymphocytes with PFOS, collapse in mitochondrial membrane potential started, but this collapse was not statistically significant (*P <* 0.05) until 4 h. PFOS significantly (*P <* 0.05) reduced mitochondrial membrane potential at two higher concentration (150 and 300 µM) at 4 h and at all concentration at 6 h in comparison with control. Cyclosporine A and BHT inhibited PFOS-induced collapse in MMP. ^*^P < 0.05, ^**^P < 0.01 and ^***^P < 0.001.

As shown in Figure 3, collapse in mitochondrial membrane potential observed after addition of PFOS to human lymphocytes but this collapse was not statistically significant (*P <* 0.05) until 2 h. PFOS significantly (*P <* 0.05) decreased mitochondrial membrane potential at all concentrations following 6 h of incubation. Pretreatment with cyclosporine A, one of the blocker of mitochondrial permeability transition (MPT) pores and BHT prevented collapse of mitochondrial membrane potential induced by PFOS in human lymphocytes.

*Lipid peroxidation*

**Figure 4:**
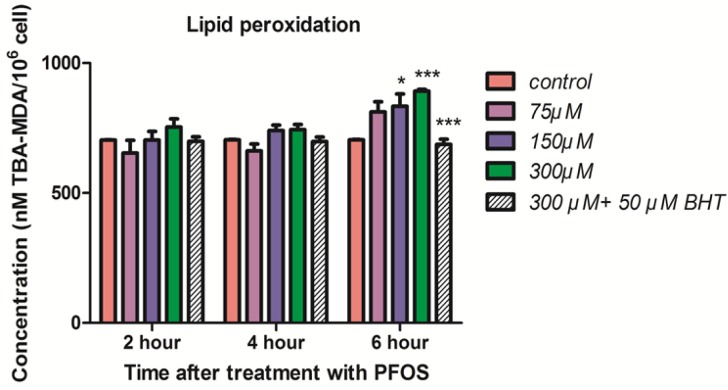
Lipid peroxidation in human lymphocyte following incubation with PFOS. Induction of lipid peroxidation in human lymphocyte after incubation with PFOS for 6 h. Lipid peroxidation was measured based on reaction of thiobarbituric acid (TBA) and malondialdehyde. After 6 h treatment, two higher concentration of PFOS (IC_50_ and 2 IC_50_) significantly (*P *< 0.05) increased MDA levels in human lymphocytes in comparison with control. BHT inhibited lipid peroxidation induced by PFOS. ^*^P < 0.05, ^**^P < 0.01 and ^***^P < 0.001

The induction of lipid peroxidation in human lymphocytes after treatment with PFOS was demonstrated in Figure 4. TBARS significantly (*P <* 0.05) increased in isolated human lymphocytes at 6 h after treatment with 150 and 300 µM PFOS. Again pretreatment with BHT inhibited raise of TBARS in human lymphocytes after treatment with PFOS.

*Proteolysis*

**Figure 5:**
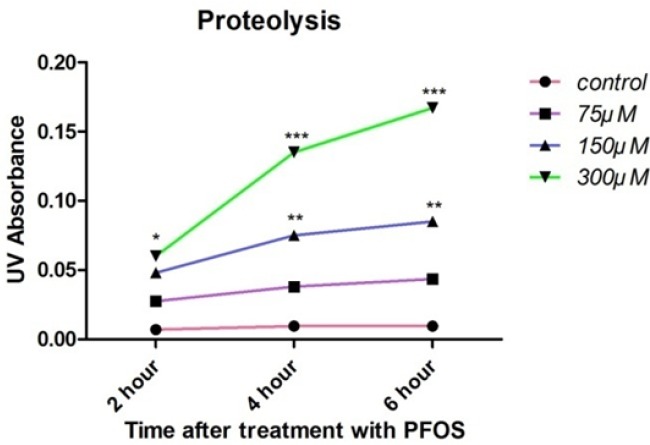
PFOS-induced cellular proteolysis in human lymphocytes.Cellular proteolysis after treatment of human lymphocytes with PFOS. Cellular proteolysis was assessed based on reaction of OPA with amino groups in presence of 2-mercaptoethanol. Statistically significant (*P <* 0.05) increase in free amino acids was observed at highest concentration of PFOS (300 µM) at all of time intervals, but 150 µM PFOS induced significant (*P <* 0.05) release of amino acids only at 4 and 6 h after treatment in comparison with control. ^*^P < 0.05, ^**^P < 0.01 and ^***^P < 0.001.

Cellular proteolysis in PFOS-treated human lymphocytes was determined based on reaction of OPA with released primary amine groups in the presence of 2-mercaptoethanol. At all time intervals, statistically significant (*P <* 0.05) rise in free amino acids was observed at highest concentration of PFOS (300 µM), while 150 µM PFOS caused significant (*P <* 0.05) release of amino acids only at 4 and 6 h of incubation (Figure 5). 

*Lysosomal membrane destabilization*

**Figure 6:**
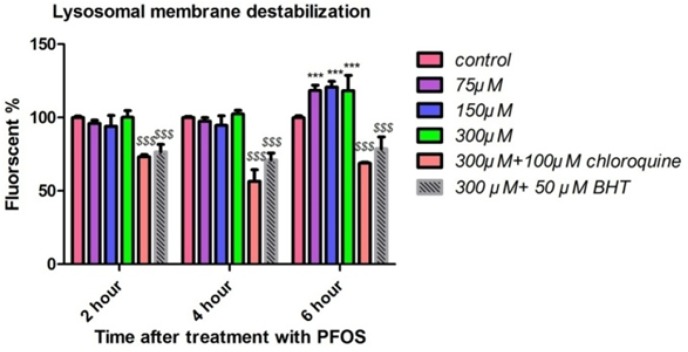
Human lymphocytes’ lysosomal membrane integrity after treatment with PFOS. Destabilization of lysosomal membrane in human lymphocytes after treatment with PFOS. Measurement of acridine orange (a tertiary amine that accumulates in lysosome) redistribution to cytosol was used as an indicator of damage to lysosomal membrane. One-hundred fifty and tree-hundred µM PFOS significantly (*P <* 0.05) induced lysosomal membrane leakage after 6 h incubation in comparison with control. BHT and chloroquine attenuated PFOS-induced lysosomal membrane destabilization.^*^P < 0.05, ^**^P < 0.01 and ^***^P < 0.001.

We measured the fluorescence of acridine orange redistributed from lysosomes in to cytosol as an indicator of lysosmal membrane leakiness in acridine orange loaded lymphocytes. PFOS caused leakage of lysosomal membrane after 6 h of incubation and this leakage was significant (*P <* 0.05) only at concentrations of 150 and 300 µM (Figure 6). PFOS induced leakage of lysosomal membrane prevented by pretreatment of chloroquine (100 µM) as an intralysosomal pH enhancer and BHT (Figure 6).

*GSH and GSSG content*

**Figure 7:**
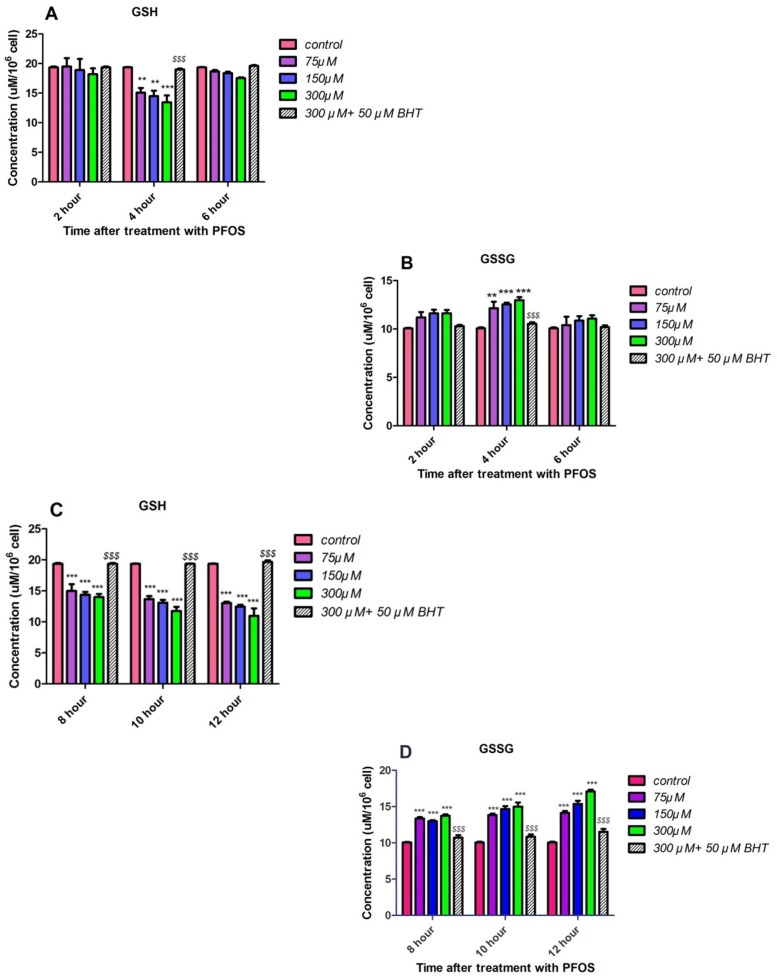
The effect of PFOS on intracellular GSH and extracellular GSSG concentrations in human lymphocytes. Intracellular GSH and extracellular GSSG concentrations in human lymphocytes following incubation with PFOS. Effect of PFOS on GSH and GSSG levels determined in accordance with Hissin and Hilf method and assessment continued until 12 h. After 6 h from the beginning of treatment, only at 4 h significant(*P *< 0.05) intracellular GSH decreaseand raises in lymphocytes extracellular GSSG in comparison with control was found as demonstrated in part A and B. As showed in part C and D constant significant increase in GSSG level and GSH collapse was observed in 8, 10 and 12 h after treatment with PFOS. BHT attenuated reduction in GSH and GSSG rises triggered by PFOS.^*^P < 0.05, ^**^P < 0.01 and ^***^P < 0.001

GSH and GSSG levels were assessed in PFOS-treated human lymphocytes based on Hissin and Hilfmethod (24). As demonstrated in Figures 7A and 7B, significant (*P <* 0.05) collapse in intracellular GSH and raise in extracellular GSSG levels was not observed until 4 h incubation with PFOS. However, alterations in GSH and GSSG levels followed until 12 h of incubation and following a considerable GSH raise and GSSG collapse at 6 h, significant (*P* < 0.05) decrease in GSH and increase in GSSG level was observed again at 8 h, and this trend remained until 10 and 12 h of treatment with PFOS (Figures 7C and 7D). Incubation with an antioxidant, BHT, prevented collapse of GSHand increase in GSSG.

*Caspase3 activity*

**Figure 8:**
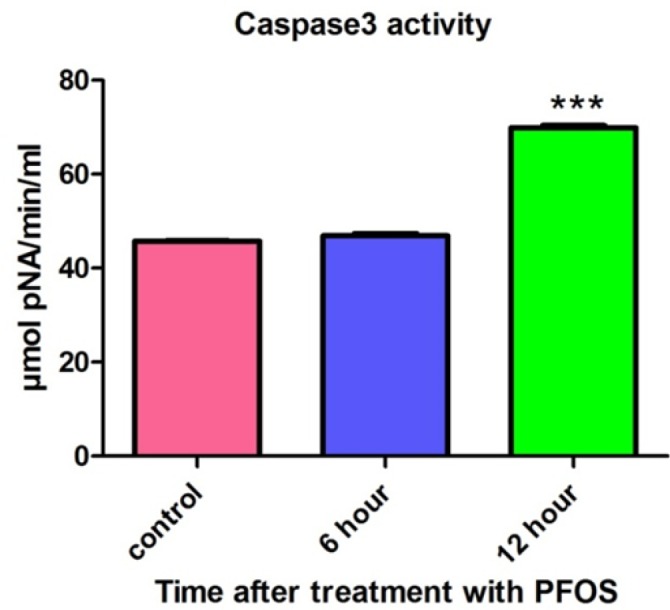
Activity of caspase-3 in human lymphocytes following PFOS treatment. The effect of PFOS on activity of caspase-3 in cultured human lymphocytes. Caspase-3 activity was assessed after treatment of human lymphocytes with IC_50 _(150 µM) of PFOS for 6 and 12 h. PFOS (150 µM), significantly (*P <* 0.05) increased activity of caspase-3 in human lymphocytes after 12 h incubation in comparison with control. ^*^P < 00.05, ^**^P < 0.01 and ^***^P < 0.001.

After treatment of human lymphocytes with PFOS, caspase-3 activity was assessed using “Sigma’s caspase-3 assay kit (CASP-3-C)”. Lymphocytes were treated with IC_50 _of PFOS (150 µM) andcaspase-3 activity was then measured at 6 and 12 h following PFOS treatment. Statistically significant (*P <* 0.05) increase in caspase-3 activity was observed in PFOS (150 µM) treated human lymphocytes after 12 h of incubation (Figure 8).

## Discussion

Perfluorinated compounds (PFCs) are applied to produce myriad consumer products. One of the major classes is the perfluorinatedsulfonates (*i.e.*, perfluorooctane sulfonic acid [PFOS]). As a result of their widespread use and the long half-life of their degradation products, PFCs have been found in the serum and tissues of humans and wildlife (5). By 2002, PFOS production was voluntarily discontinue by its major manufacturer. Although these voluntary measures have decreased release of PFOS and PFOA into the environment, the stability of these compounds makes certain that they will have a persistent presence in environmental and biological media.

The first investigation that described the immunotoxicity of PFOS was published in 2007 (25). In this study, decrease in lymphoid organ weights, lymphoid cell counts, and antibody synthesis were demonstrated after oral (dietary or gavage) exposure. Animal experiments indicated that PFCs can negatively affect immune function and there are evidences indicating that PFOS causes cell death in human lymphocytes. At first we assessed the cytotoxicity of PFOS in human lymphocytes after treatment with a wide range of PFOS concentrations.Our result demonstrateddose-dependent reduction in lymphocyte viability after 12 h treatment.

It has been reported in several studies that cytotoxicity of PFOS is closely related to generation of ROS and induction of oxidative stress. For example in study doneby C. Wan *et al.* it was showed that PFOS induced ROS formation in HepG2 cells in a dose-dependent manner. However, after 12 h of treatment, the ROS generation reached its peak and after 24 h exposure collapse in ROS formation was observed (26). Zhang *et al. *measured generation of ROS in thymocytes and splenocytes isolated from male C57Bl/6 mice following treatment with PFOS using 100 μM DCFH-DA and the Becton Dickinson flow cytometer. Significant increase in generation of ROS was observed in both spleen and thymus following 7 days of oral exposure to ≥5 mg PFOS/kg/day (27). In this study we also demonstrated that after 4 h incubation, PFOS induce ROS formation in human lymphocytes followed by a collapse in 6 h, but when we assessed ROS generation at times after 6 h, constant rise in ROS generation was again observed. When we pretreated lymphocyte with an antioxidant, BHT, ROS formation inhibited in our cells. In our research we not only detected the increased ROS formation in human lymphocytes which are quite different from mouse thymocytes and splenocytes, but also we further elucidated the case and found a mechanistic and functiononomic relation between ROS increase and subsequent membrane damage in both mitochondria and lysosomes leading to initiation of apoptosis signaling through mitochondrial membrane depolarization definitely confirmed by measurement of caspase-3 activation, the final mediator of apoptosis, and also cellular proteolysis initiated from lysosomal membrane leakiness and release of lysosomal proteases. Overproduction of ROS can overwhelm cellular antioxidant capacity and subsequently cause damage to cellular macromolecules (such as DNA and proteins) and cell membrane. We observed collapse of intracellular GSH and increase in extracellular GSSG after treatment of human lymphocytes with different concentration of PFOS. In other study that C. Liu *et al.* investigated the effect of PFOS on primary cultured hepatocytes of fresh water tilapia (*Oreochromisniloticus*), PFOS caused significant depletion of glutathione (28), such results were also observed by other researchers. Y.H. Zhang *et al.* treated adult male C57Bl/6 mice with PFOS for 7 days and activities of anti-oxidative enzymes (GPx) and the GSH levels in splenocytes isolated from exposed mice were significantly decreased (27). Glutathione is the most important antioxidant molecule in cell and glutathione depletion that happen following generation of significant level of ROS, causes considerable damage to lymphocyte membrane due to lipid peroxidation. Interestingly, in this study MDA, one of the byproducts of lipid peroxidation significantly increased after 6h incubation of human lymphocytes with PFOS in comparison with control lymphocytes. On the other hand, pretreatment of lymphocytes with an antioxidantBHT prevented increase in TBARS production. Other studies also reported significant increase of lipid peroxidation in different cells treated with PFOS. Z. Mao *et al.* measured the intracellular MDA level in lung cancer A549 cells after 24 h treatment with PFOS (0–200 mM) and significant dose-dependent raise in MDA levels wasdemonstrated (6.1, 37.8, 80.4, and 90.3% compared with untreatedcontrol) (29).

Formation of ROS can happen in different sub-cellular organelles but the most common source of ROS formation inside the cells are mitochondria, which are responsible for aerobic respiration (30). In addition, mitochondrion can be adversely affected by oxidative stress. In several studies mitochondria was mentioned as a subcellular target for PFOS. Furthermore, the collapse of mitochondrial membrane potential (damage to mitochondria) was suggested as a mechanism for PFOS toxicity in different cell lines (31, 32). To investigate the apoptotic effects of PFOS on microglia as the brain’s innate immune system, Zhang *et al.* used murine N9 cell line as a model for their research. Effect of PFOS on MMP in N9 cells measured by using Rh-123 and results showed association between PFOS-induced significant cell viability reduction and collapse in mitochondrial membrane potential (33). In accordance with previous studies, regarding the connection between PFOS-induced cell death in human lymphocytes and collapse of mitochondrial membrane potential (as an index for mitochondrial damage) was demonstrated in our study. Inhibition of mitochondrial membrane potential collapse by cyclosporine A and BHT in PFOS treated human lymphocytes, implies the possible role of ROS generation in PFOS-induced toxic effects on mitochondria. Mitochondrial damage finally leads to opening of Mitochondrial Permeability Transition (MPT) pores that in turn allowed the release of cytochrome c from mitochondria in to cytosol. Cyt c is an essential component of the electron transport chainthat when it is released in to cytosol, causes dysfunction of mitochondrial energy generation process and activation of caspases that eventually lead to cell death. To assess the possible role of caspases pathway in PFOS toxicity toward human lymphocytes, the activity of caspase3 the final mediator of apotosis was also measured in this study and the involvement of this apoptosis signaling in PFOS-induced lymphocyte cytotoxicity was also proved. Consistent with this result, relation between caspases pathway and PFOS induced apoptosis has been shown in several studies that investigated toxicity of PFCs in different cellular models (31, 34). For example, X. Wang *et al.* demonstrated an increase in caspase-3/7 activity following exposure of human–hamster hybrid (A_L_) cells to 200 µM PFOS for 1 day. In addition caspase-3/7 activity was significantly reduced after co-treatment with 0.5% DMSO, 50 U/mL PEG-SOD, and 0.2 mML-NMMA (NG-methyl-l-arginine) (32). These results provided strong evidence for the role of ROS/RNS in PFOS mediated caspases activation and cell death. 

It has been shown that treatment of HepG2 cells with 150 and 200 µM PFOS for 12 h led to a severe concentration-dependent decrease in lysosomal membrane integrity (reduction of red puncta (intact lysosome) after staining with AO) compared with the control. In this study, it has also been found that PFOS reduce the expression of LAMP-2 (an integral membrane proteins to maintain lysosomal structural integrity and function) in HepG2 cells, which might decrease the stability of lysosomal membrane (35). In accordance with this study our results also showed relocation of acridine orange from intact lysosome to the cytoplasm following treatment of acridine orange loaded human lymphocytes with PFOS. Furthermore, pretreatment of these cells with an endocytosis inhibitor, chloroquine (100 µM) and an antioxidant, BHT prevented leakage of lysosomal membrane showing close relation between ROS Production and damage to lysosomal membrane. Destabilization of lysosomal membrane may lead to release of lysosomal proteases(*e.g. *cathepsins) that in turn can activate caspases cascade through mitochondrial targeting and release of cytochrome C which finally orchestrates lymphocytecell death signaling (36). As shown in our study, leakage of lysosmal membrane and release of lysosomal enzymes can also lead to proteolysis that is one of the eventual events in cellular cytotoxicity.

## Conclusion

Based on our results we conclude that environmentally related concentration of PFOS can causes cytotoxic effect in human lymphocytes. Human lymphocytes death and following suppression of immune system may be through PFOS induced oxidative stress events such as glutathione depletion and lipid peroxidation, and furthermore damage to cell organelles such as mitochondria and lysosome. In addition involvement of cellular proteolysis and activation of caspases pathway was proved in PFOS cytotoxic effect toward human lymphocytes. Taking together, these considerable additional findings in human cells exposed to PFOS, providing evidence that all of them are mechanistically related together in a functiononomic manner make our research novel.
